# The medical care of patients with primary care home nursing is complex and influenced by non-medical factors: a comprehensive retrospective study from a suburban area in Sweden

**DOI:** 10.1186/1472-6963-4-22

**Published:** 2004-08-26

**Authors:** Sonja Modin, Anna-Karin Furhoff

**Affiliations:** 1 Centre for Family Medicine, Department of Clinical Sciences, Karolinska Institutet, Alfred Nobels allé 12, S-141 83 Huddinge, Sweden

**Keywords:** home nursing, home health care, home care, hospitalisation, outpatient visits, health care utilisation, primary health care, specialised medical care

## Abstract

**Background:**

The reduced number of hospital beds and an ageing population have resulted in growing demands for home nursing. We know very little about the comprehensive care of these patients. The objectives were to identify the care, in addition to primary health care, of patients with primary-care home nursing to give a comprehensive view of their care and to investigate how personal, social and functional factors influence the use of specialised medical care.

**Methods:**

One-third (158) of all patients receiving primary-care home nursing in an area were sampled, and 73 % (116) were included. Their care from October 1995 until October 1996 was investigated by sending questionnaires to district nurses and home-help providers and by collecting retrospective data from primary-care records and official statistics. We used non-parametric statistical methods, i.e. medians and minimum – maximum, χ2, and the Mann-Whitney test, since the data were not normally distributed. Conditional logistic regression was used to study whether personal, social or functional factors influenced the chance (expressed as odds ratio) that study patients had made visits to or had received inpatient care from specialised medical care during the study year.

**Results:**

56 % of the patients had been hospitalised. 73 % had made outpatient visits to specialised medical care. The care took place at 14 different hospitals, and more than 22 specialities were involved, but local care predominated. Almost all patients visited doctors, usually in both primary and specialised medical care. Patients who saw doctors in specialised care had more help from all other categories of care. Patients who received help from their families made more visits to specialised medical care and patients with severe ADL dependence made fewer visits.

**Conclusions:**

The care of patients with primary-care home nursing is complex. Apart from home nursing, all patients also made outpatient visits to doctors, usually in both primary and specialised medical care. Many different caregivers and professions were involved. Reduced functional capacity decreased and help from family members increased the chance of having received outpatient specialised medical care. This raises questions concerning the medical care for patients with both medical and functional problems.

## Background

This article describes the situation in a suburban area in Sweden, and concerns the comprehensive medical care of patients with primary-care home nursing, i.e. patients who require regular assistance from nurses in their homes in order to maintain their health and manage their health problems. This does not include hospital-at-home care, where a multiprofessional team is responsible for the medical care.

The use of inpatient hospital care in Sweden as in many other Western countries has diminished in recent decades. In combination with an ageing population with greater needs for medical care, this has resulted in an increase in different forms of home care. The overall situation is similar in many countries, although local conditions may vary considerably [1-4].

The shift of care from the hospital to the home "has had an enormous impact on care recipients, their families and friends, and in-home service providers." [3] and "is changing the meanings, material conditions, spatio-temporal orderings and social relations of both domestic life and health-care work." [5]. One effect has been "the fragmentation and dispersion of specialised health services from hospitals to alternative locations.".. "especially revealed for people with diminished mobility" [4]. These changes make the care of patients with home nursing an important area for investigation from the perspectives both of patients and of health care workers.

Much is known about the medical care, especially inpatient and outpatient hospital care of older persons, and that factors like age, health problems and socio-economic conditions influence the utilisation of inpatient hospital care [6,7]. Hospital-at-home care has also been the subject of several studies [8-10]. However, most investigations of home care and home nursing, concern care in the patient's home [11,12]. Other studies have focused on home nursing and medical care of patients with specific diseases, and whether care at home influences the risk of rehospitalisation [13,14]. But little is known about the total care of patients with home nursing, which not only includes what takes place in the home, but also the care received in hospitals, out-patient departments, and from doctors in general practice, etc. There is also a lack of knowledge about what factors influence the care of these patients, apart from medical necessities. In view of the expansion of this health care sector, such knowledge should be of value, particularly for planning purposes.

In a previous paper [15] we have presented a picture of the patients with home nursing, in a Swedish suburb, and the primary health care of those patients. The patients were old (median age 83 years, range 46–95), and many had functional problems (e.g. reduced mobility (50%), vision (46%), cognitive ability (33%), hearing (29%)), and symptoms (e.g. musculoskeletal pain (53%), fatigue (46%), anxiety (44%)). Cardiovascular disorders (42%), psychiatric disorders including dementia (27%), and musculoskeletal disorders (21%) were the most common diagnoses in family physician records. Most patients had several symptoms and more than one diagnosis. All had contact with district nurses or assistant nurses, usually once a week or every second week. Ninety-seven percent of the patients had had medical care initiated by their family physician during the study year, but much of the care was carried out without direct contact between family physician and patient, as a significant part of the medical care was performed through collaboration with district nurses. This meant that the patients with home nursing saw their family physicians less often than other patients of comparable age.

In view of the lack of knowledge in this expanding field, we considered it important to investigate and describe the comprehensive care of patients with home nursing as a starting point for further research and development.

The objectives of the present paper are as follows:

To identify the specialised medical care of patients who were receiving primary-care home nursing.

To give a comprehensive view of the care of these patients.

To investigate how personal, social and functional factors and help by relatives influenced the use of specialised medical care by these patients.

## Methods

The study was performed in a suburban area of Stockholm with 40 000 inhabitants, 18 percent of whom were 70 years of age or older. The care of patients, with primary-care home nursing, who lived in ordinary houses or flats was studied. Patients in the study area who received regular nursing care at home for a period of more than two weeks from the district nurses in primary health care were registered as primary-care home nursing patients (Table 1).

In Stockholm, patients can choose to go to any of the hospitals (11 emergency hospitals and several smaller geriatric hospitals during the study year) or outpatient departments in the city. Two emergency hospitals, two geriatric hospitals and two wards for psychiatric inpatient care were located in or close to the study area.

During the registration week (21 to 27 October 1996), 486 patients in the study area had primary-care home nursing. Using a random table, we selected one-third of the patients of each district nurse for the study.

The study was designed as a retrospective study of the comprehensive medical care of patients with primary-care home nursing, including the medical care they received both in their homes and in other places. It was also designed to study whether non medical factors that are common among patients with home nursing may have influenced the care. The data were not obtained from the patients, but from records and from the family physicians and district nurses responsible for the patients' primary health care, i.e. professionals dealing with the patients' medical problems. Data were also obtained from the official statistics of the Swedish County Councils, a source that is generally considered by researchers to be of high quality and well suited for scientific purposes. A description of the patients and the care performed by family physicians, district nurses and assistant nurses was given in a previous article [15].

The hypotheses were

- that care outside the home comprises a substantial part of the total care of patients with home nursing.

- that personal, social and functional factors may influence the use of medical care outside the home

Information concerning the situation during the registration week was obtained from questionnaires distributed the week following the registration week. Retrospective data from the study year (28 October 1995 to 27 October 1996) were obtained from the official statistics, and the family physician (21 family physicians) and nursing (20 district nurses) primary health care records.

The official statistics were used to identify and describe inpatient care and outpatient visits in specialised medical care, and the number of home and practice visits to/by the nurses in primary health care. Notes in the family physician records were used to describe the family physician care (reason for contact, who had been in contact and measures undertaken) as well as the diagnoses of the patients [16]. Notes in the nursing records were used by the district nurses to identify nursing procedures. The protocol for extraction of information from the nursing records comprised 18 questions with fixed-alternative answers and was designed for the study by the Stockholm Gerontology Research Centre in cooperation with a group of district nurses [17].

Personal, social and functional data concerning the situation during registration week, as well as patients' symptoms, and help from relatives at that time, were obtained from the questionnaires. When possible, validated questions that had been tested in other studies were used, but some were modified to suit the situation in home nursing. These revisions were made by the Stockholm Gerontology Research Centre in cooperation with a group of district nurses [17]. Personal and social factors included age and sex, whether or not the patient lived alone, and whether there were one or more relatives who assumed responsibility for a substantial amount of the care. Functional problems concerned cognitive function (difficulty knowing the day of the week, finding the way home and/or recognising relatives/caregivers), mobility (being unable to move about in the immediate surroundings), and ADL capacity. For ADL capacity the different Katz index functions were used and the patients were grouped according to the degree of ADL dependency. Patients in *Group 1 *were either without functional deficiencies or dependent only regarding cleaning, shopping and/or transport, patient in *Group 2 *were, in additions, dependent with respect to cooking, bathing and/or dressing, but not eating, and patients in *Group 3 *were in addition dependent concerning eating. The factor mobility was excluded from the ADL groups, as this was assessed separately using data from other questions. The factors toileting and continence were excluded, as the answers were not consistent when compared with answers to questions concerning the same functions in other parts of the questionnaire.

Symptoms were registered in a 23-item protocol originally used in studies of nursing homes, but modified to suit the situation in home nursing. The questionnaires also included questions about how long the patient had had home nursing, and whether the patient had had contact with any private doctor in specialised medical care.

The questions were thus chosen so that the district nurses responsible for the care could answer them without additional patients assessments, either because the information would be well known to them since they were responsible for the nursing care or because the answers were based on assessment tools used in the regular care, such as that used for ADL.

Questionnaires sent to the home-help providers focused on whether the patient had home help (Table 1).

From the randomised one-third of selected patients (n = 158), we excluded 42, (16 declined to participate, and for ethical reasons we did not inquire why; ten had been discharged from home nursing, died or had been admitted to hospital; four could not participate for other reasons). Information about all patients from one district nurse was excluded (12 patients), since it was obvious that she had not understood the questions, leaving 116 patients (73%).

Information could not be obtained from all sources for all patients. Visits to private doctors in specialised medical care and to private physiotherapists are not included, as they were not included in the official statistics. According to questionnaire responses from the district nurses, 11 patients saw private doctors in specialised medical care on a regular basis. We have no information about private physiotherapists.

As the number of visits and care periods did not have a normal distribution, we used non-parametric statistical methods, i.e. medians and minimum – maximum. The Mann-Whitney test and χ2 were used to compare differences between groups. Conditional logistic regression was used to study whether personal, social or functional factors influenced the chance (expressed as odds ratio) that study patients had made visits to or had had inpatient specialised medical care during the study year. We used a case control design where patients with specialised care were cases and patients without this care were controls. The different factors were first tested by univariate logistic regression. The factors that showed significant influences were included in a multiple logistic regression model. One of these factors (home help) showed no significant influence when included in the model, and it was therefore excluded from the main effect model [18]. The factors without significant influence were also tested and included in the model, but no significant influence was found. The SPSS data-analysing system, version 11.0, was used for the analyses.

The study was approved by the Research Ethics Committee at Huddinge University Hospital. This approval included the design of the study as well as the way informed consent was obtained from the individual patients.

## Results

Eighty-nine of the 116 study patients (77%) lived alone, 86 patients (74%) were women and 41 (35%) received substantial assistance from family members according to the district nurses. Sixty-eight patients (65%) had home help. Patients who lived alone were less likely to have assistance from family members than those who lived with a relative (31% compared to 58%, p < 0.05), but on the other hand they were more likely to have home help (76% compared to 29%, p < 0.001). Patients who got help from family members were less likely to have home help (51% compared to 75%, p < 0.05).

### Specialised inpatient care

More than half of the patients had been admitted to hospital during the study year (Table 2). Among these, the majority were admitted twice or more and spent more than three weeks in hospital. In all, more than 15 specialities were represented. Geriatric care represented almost 2/3 of all days in inpatient care, but the care by specialists at the different emergency hospitals involved more patients and a greater number of care periods. The most common specialities were general internal medicine (32 study patients and 29% of the care periods), surgery (10 study patients and 6% of the care periods), neurology (nine study patients and 5% of the care periods) and orthopaedics (seven study patients and 4% of the care periods). Almost all inpatient care periods (93%) were spent at the two emergency hospitals, the two geriatric hospitals, and the two local psychiatric wards located in or close to the study area, but six other emergency hospitals and one other geriatric hospital also provided care for these patients.

The median values for all study patients were one care period and four days in care. The average number of care periods and days in inpatient care in Stockholm for the persons 75 to 84 years of age, and 85 years of age or older, were 0.6 of a care period and five days in inpatient care and 0.8 of a care period and seven days in inpatient care, respectively [19].

### Specialised outpatient care

During the study year a majority of the patients made outpatient visits to hospitals and outpatient departments (Table 3). In all, 22 different specialities were represented. Visits to departments of general internal medicine were most common (46% of the study patients, 22% of all visits), followed by visits to departments of surgery (26% of the study patients, 6% of the visits), orthopaedics (22% of the study patients, 6% of the visits) and ophthalmology (21% of the study patients, 9% of the visits). The visits in specialised care took place at eight different emergency hospitals, two geriatric hospitals, and two local psychiatric and one local ophthalmology department. However, 86% of the visits were to the two emergency hospitals located in or close to the study area. Of the 80 patients who made outpatient visits to hospitals 46 percent visited more than one speciality.

The median value for visits to doctors in specialised care for all study patients was one visit. The average numbers of outpatient visits to doctors outside primary health care (excluding doctors in private practice) in Stockholm for persons 75–84 years of age and 85 years of age or older were 1.7 and 0.8, respectively [20].

### The comprehensive view

In the 104 cases where a family physician record was found, a visit to either their family physician or a doctor in outpatient specialised care, or both, was found for 95 patients (91%) (median 3 visits). Approximately one third (32 %) of the patients saw doctors from two or more specialities, GP care not included. Thirty-eight study patients (33%) had visited a physiotherapist or an occupational therapist (290 visits in all). Of these, 141 visits (49%) took place in primary health care and comprised 23 (20%) of the study patients (Table 3). Doctors and physiotherapists in private practice are not included.

More detailed examples of the care picture on an individual level are given in Table 4, where the problems and the medical care provided for two patients are described. The objective is to give a picture of what the care may look like from the perspective of the individual patient, as well as to present a picture of what conditions may be like for the different caregivers with the task of cooperating and coordinating the care. We have chosen one patient with three visits to specialised care, which was the median number among those who had specialised care, and one patient with many visits to specialised care. In both cases, it was possible to obtain data from all sources of information.

Patients who did not see a family physician during the study year did not receive significantly more or less care from other caregivers (Table 5). Patients with visits to doctors in specialised care made significantly more visits to family physicians, saw the district nurses and other caregivers in specialised care more often, and also had more inpatient care. Patients who had been admitted to the hospital had more contacts with both doctors and other caregivers in outpatient specialised care, but not with the family physicians or the nurses in primary health care.

### The influence of personal, social and functional factors

The influence of personal, social or functional factors on the chance of receiving specialised medical care was tested (Table 6). When the groups with different degrees of ADL dependency were tested, we found no difference between groups 1 (30 patients) and 2 (62 patients), but found that both groups 1 and 2 differed from group 3 (23 patients). Therefore groups 1 and 2 were combined into one group (1–2) and compared with group 3 in the model. Patients with severe ADL dependence (group 3) were less likely, and patients who had help from family members were more likely to make outpatient visits in specialised medical care. The same factors were tested to see if they influenced the chance that a patient had had inpatient care, but here we found no significant association.

## Discussion

A majority of the patients with primary-care home nursing also received both inpatient and outpatient specialised medical care. Patients received care from home help, family members, family physicians, nurses in primary health care, and doctors and other caregivers in specialised medical care, thus providing a very complex picture. Many different hospitals and specialities were involved. Almost all patients had seen one or more doctors during the study year. Those who saw doctors in specialised care had more help from all categories of medical care. Patients with severely reduced ADL capacity were less likely, and patients with help from family members were more likely to have made outpatient visits in specialised medical care.

The limitations of this paper include that personal, social and functional factors as well as symptoms were recorded by the district nurses and not reported directly by the patients, which means that this is second hand information, sometimes including an assessment. However, all of the participating district nurses had four and a half years of university education as is required, as well as many years of professional experience, which should ensure a sound basis for their assessments. Most of the personal and social factors were of the kind that are readily available concerning any patient and do not need assessments. The exception is participation of family members in the care, where the district nurse with her often longstanding contact with the patient and family is a suitable source of information. The functional factors in the Katz ADL index, mobility and cognitive function, are functions the nurses were used to assessing and reporting in their cooperation with hospitals and home help services. The only inconsistencies found concerned toileting and continence, which were consequently excluded. Further, symptoms could have been underestimated, as the district nurses would only be aware of them if they were obvious, or if the patient reported them, but they have only been used to describe patients, and not for any analysis.

The study presents the medical care of patients with primary-care home nursing in one suburban area in one country. There are differences between the health-care systems and the informal and formal networks of care in different countries and sometimes also in different areas in the same country. This means that our conclusions are valid for patients with primary-care home nursing in this area; verifying whether they are valid in other areas would require further studies. However, as the study patients have the same type of problems described in other studies of patients with home nursing [21,22], their care needs are probably not different from those of patients receiving home nursing elsewhere.

We have studied the situation in a city with easy access to near-by, specialised care, and with many different hospitals where patients are free to obtain medical services without a referral. This will have increased the number of hospitals and units involved in the care, as well as the number of outpatient visits [23,24]. This part of our results might be representative for other areas with easily accessible specialised care, but to confirm this would require further studies.

The strengths of this paper include the following. A randomised third of a population is considered to provide a sufficient basis for conclusions about all patients receiving primary-care home nursing in a population, and a participation rate of 73 % is acceptable. The patients who were excluded were of the same age (median age 83, range 42–98 years) as the participants but might have had more severe conditions, as patients who had died or were admitted to hospital during the registration week were excluded. These patients probably did not receive less medical care than those who were included. Information about a few patients was missing from some sources. All practices had roughly the same proportion of drop-outs and missing data, and the reasons can be considered to be random. The results should then be valid for all patients with primary-care home nursing, in the study area. As the patients are similar to those with home nursing described in other studies, this study may serve as a reference for further investigation of the care of patients with home nursing.

These patients who had primary-care home nursing also had many instances of specialised inpatient and outpatient medical care. However, their care did not differ substantially from that of the average population of comparable age in Stockholm County during 1996 [19,20]. This is somewhat surprising, as these patients with health problems could be expected to use more medical care. On the other hand, patients who regard a primary-care physician as their personal physician rather than a doctor in specialised care have lower total health-care expenditures [25], and patients who identify a doctor outside the hospital as their primary source of care are hospitalised less often [26]. Practice populations also have a major reduction in hospital care when a nurse is introduced into the primary-health-care team [27]. Since only patients with primary-care home nursing were included in the study and almost all (97%) received medical care from a family physician, this may explain why they did not use more specialised care than the average population.

There was one group with significantly more medical care than the others, i.e. those patients who made outpatient visits to doctors in specialised care. The probable reason is that they had more severe medical conditions, but this is not possible to confirm in the present study.

Inpatient care seemed to be strongly associated with more outpatient contacts in specialised care, but not with more contacts in primary health care. It is not possible to say whether this was the result of a greater need for specialised care, or the result of a tendency to keep the patients in specialised care [28].

Patients with no visits from a family physician received neither more nor less medical or nursing care elsewhere. The rest of the care was apparently not influenced by the low priority this was given by the family physicians.

The care of an individual patient often included several caregivers from different organisations. For the individual patient who meets many different persons and goes to many different places, this means a lack of continuity. For this group of elderly patients with complicated medical problems, reduced mobility, reduced cognitive ability and reduced ADL-capacity, the risk that the individual caregiver does not have the proper information when decisions are made must be extremely great, especially taking into account the problems involved in the exchange of information between primary care and specialised care [28]. On an organisational level, many different caregivers make demands on systems and time for co-operation and the exchange of information. From a health care perspective the risk of inefficiency, low quality and/or high costs is evident, as at one end the same procedures might be done by several caregivers, while at the other end vital measures might not be performed because no one has the total picture. Several Swedish studies describe serious quality problems related to the medication of persons in home nursing, and these may partially reflect this situation [29,30], while other studies describe the lack of co-ordination and of an overall picture in the care of old persons with multiple problems [31,32].

In Sweden, the share of health care expenditures allocated to primary health care is often low (12 – 21 %) compared to other parts of Western Europe, where it is frequently 20 percent or more [33]. Further, this figure has not increased to any great extent since 1980, even though the inpatient hospital care has decreased dramatically [33-35]. The picture we report, where many patients remain in specialised care, is compatible with this economic situation.

We found that functional and social factors influenced the chance of a patient having made outpatient visits to specialised medical care. Severe ADL dependence reduced the chance and receiving help from family members increased the chance. The fact that patients with severe ADL dependence do not make many outpatient visits is not surprising as reduced function limits the possibility to get to locations outside the home. The findings that help from family members increased the chance of having made outpatient visits to specialised medical care is not surprising either, in view of the age and functional problems of the patients. Relatives might be more observant concerning new symptoms, might easier establish contact and assist in transportation of the patients, than professional caregivers. Is the care organised so that old patients with multiple diseases and reduced functions need the help of a relative in order to get outpatient specialised medical care? Or do patients who get help from family members have more severe medical problems, even though there are no differences in primary-health-care diagnoses? Further studies are needed in this area.

## Conclusions

The picture that evolves from our study is that the care of patients with home nursing is more complex than has previously been assumed. In parallel with their primary-care home nursing, all patients had contact with doctors, often from both primary and specialised medical care. The situation resembles that in a hospital ward, where many different caregivers and many different professions are involved in the care of the same patient, but without the ward's geographical and temporal unity. This renders it almost impossible for the individual patient to get continuity in all aspects of care, and the possibility of cost effective care of good quality is diminished.

Patients with home nursing have complicated medical problems and both nurses and doctors are involved in their care, contrary to the previous belief that some patients are cared for by nurses alone. Instead, there seems to be one group of patients with home nursing who also need both primary and specialised care, and who have greater care needs than other patients with home nursing.

That reduced function decreases and help from family members increases the chance of getting outpatient, specialised medical care raises questions concerning the medical care for patients with both medical and functional problems.

Our conclusions are based on a study in one suburban area, and further studies are needed in order to confirm them.

## List of abbreviations used (if any)

ADL Activities of Daily Living

ENT Ear Nose and Throat

## Competing interests

None declared.

## Authors' contributions

SM designed and carried out the study, performed the statistical analysis, participated in the interpretation of results, wrote the initial draft of the manuscript and made subsequent revisions. AKF participated in the interpretation of results and made critical revisions of the manuscript. Both authors have read and approved the final manuscript.

## Pre-publication history

The pre-publication history for this paper can be accessed here:



## Figures and Tables

**Table 1:** Definitions used in the article

Home help	Persons who deliver general care at home. They do not necessarily have medical training
Home-help organisation	The organisation that delivers home help services
Home-help provider	Decide whether a person can get subsidized home help, and if so how much.
Hospital-at-home	Medical care in the home performed by a multi-professional team. Patients with this care were not included in this study, but some patients had had this form of care during the study year.
Nurses in primary health care	District nurses and assistant nurses working in primary health care.
Primary-care home nursing	Home nursing that is part of primary health care, and is performed by district nurses or by assistant nurses under the supervision of the district nurse. The family physician can be of support or actively involved in the care. Patients with this form of care were included in the study if they lived in ordinary houses or flats.
Specialised medical care	Medical care outside of primary health care, often performed at hospitals or in outpatient clinics.

**Table 2:** Inpatient care and hospital-at-home care. Information from the official statistics of Stockholm County for the study year for the patients (n = 116) receiving primary-care home nursing (information missing for one patient).

	No. of patients		No. of care episodes in patients with inpatient or hospital-at-home.			No. of care days in patients with inpatient or hospital-at-home.		
Type of care	n	%	Total number	Median	Min – max	Total number	Median	Min – max
All forms of inpatient care	64	56	190	2	1–10	1799	23	1–138
Inpatient care from emergency hospital specialities^1^	51	44	107	1.5	1–10	599	7.5	1–49
Geriatric inpatient care	42	37	77	1	1–6	1063	19	1–109
Psychiatric inpatient care	2	2	3	-	1–2	87	43.5	19–68
Hospital-at-home	3	3	3	-	1–1	50	10	2–38

^1 ^E.g. internal medicine, surgery, neurology, orthopaedics

**Table 3:** Outpatient medical care. Information from the official statistics of Stockholm County for the study year for the patients (n = 116) receiving primary-care home nursing (information missing for one patient).

		Patients		No. of visits by those who made visits		
Type of care	Type of caregiver	N	%	n	Median	Min – max
All forms of specialised care	All professions	83	72	957	5	1–173
	Doctor	80	70	356	3	1–23
	Physiotherapist or occupational therapist	18	16	147	5.5	1–33
	Other^1 ^or unknown	60	52	454	2	1–166
Emergency hospital specialities^2^	All professions	78	68	785	5	1–173
	Doctor	76	66	322	2.5	1–23
	Physiotherapist or occupational therapist	7	6	52	4	1–23
	Other^1 ^or unknown	52	45	411	2	1–166
Geriatric speciality	All professions	18	16	168	5	1–45
	Doctor	15	13	34	2	1–8
	Physiotherapist or occupational therapist	13	11	95	4	1–33
	Other^1 ^or unknown	12	10	39	2.5	1–9
Psychiatry	All professions	2	2	4	-	2–2

^1 ^E.g. nurse, welfare officer, speech therapist, dietician, ^2 ^E.g. internal medicine, surgery, orthopaedics, ophthalmology, dermatology All forms of care includes care at emergency hospital specialities, geriatric care, and psychiatric care. All professions includes doctors, physiotherapists and occupational therapists and other or unknown caregivers

**Table 4:** Examples of care situations during the study year involving patients receiving primary-care home nursing.

82-year-old woman who lives alone, has no home help, has help from family members. (Many visits in specialised care)
Main medical problems: Asthma, facial skin disease, polyposis of the nose, needs a hearing aid, cataract operation, urinary problems, fever, mammary problems. Functional problems: Reduced vision, reduced hearing. Primary-care home nursing for 5 months.
Primary health care during the study year: 159 visits by nurses, who helped to prepare inhalations and made medical assessments.
The family physician record shows 2 notes concerning visits and 2 notes without a visit. Diagnoses in family physician record: Fever, skin disease, pathological urinary sample, examination including mammography.
Medical care outside of primary health care: 18 visits (3 to audiology, 1 to ENT, 2 to dermatology, 8 to ophthalmology, 2 to surgery, 3 to pulmonary medicine). 14 of these visits were to doctors. One period of inpatient care: 2 days in the ENT clinic.

88-year-old woman who lives alone, has home help. (Three visits in specialised care)
Main medical problems: Heart failure, atrial fibrillation, depression, infections. Functional problems: Reduced vision, reduced hearing, reduced mobility and reduced cognitive function. Primary-care home nursing for one year.
Primary health care during the study year:52 home visits by nurses, who dispensed tablets and helped with general care, social rehabilitation, preparation before tests, blood and urinary sampling, measuring of blood pressure, and made medical assessments and did care planning.
The family physician record shows 3 notes concerning home visits, 3 concerning practice visits and 9 notes without a visit. Diagnoses in the family physician record: Depression, heart failure, atrial fibrillation, upper respiratory tract infection, pneumonia, cystitis.
Medical care outside primary health care:2 visits to ophthalmology, 1 visit to general internal medicine. One visits was to a doctor. 3 periods of inpatient care in geriatric clinic, 18 days.

**Table 5:** Number of visits to principal caregivers. The number of visits are given in relation to the presence or absence of visits to family physicians and to doctors in specialised medical care, and to the presence or absence of inpatient care during the study year, for patients with primary-care home nursing (n = 116; information is missing concerning family physician visits for 12 patients, for specialised care for one patient). Number of patients (n), median (M), and p-value (Mann-Whitney test)

			No. of visits to family physicians		No. of visits by district nurses		No. of visits to doctors in specialised care		No. of visits to caregivers other than doctors in specialised medical care		No. of inpatient care periods	
		n	M	p	M	p	M	p	M	p	M	p

Visits to family physician	No	21	-	-	29	n.s.	1	n.s.	1	n.s.	0	n.s.
	Yes	83	-		36		1		0		1	

Visits to doctors in	No	35	1	< 0.01	25	< 0.05	-	-	0	< 0.001	0	< 0.001
specialised medical care	Yes	80	2		37		-		2		2	

Inpatient care	No	51	1	n.s.	34	n.s.	1	< 0.001	0	< 0.001	-	-
	Yes	64	2		36		2		1		-	

**Table 6:** The association of non-medical factors to outpatient visits in specialised care. Personal, social and functional factors concerning the patients receiving primary-care home nursing and how they are associated with the chance (odds ratio) of making outpatient visits in specialised medical care during the study year. (n = 115). Logistic regression.

			Univariate		Main effect model	
Factors		N	Odds ratio	95% Conf. Interval	Odds ratio	95% Conf. Interval

Age	> 83 years of age	53	0.68	0.30–1.53		
	≤ 83 years of age	62	1	(Reference)		
Sex	Male	30	1.37	0.52–3.60		
	Female	85	1	(Reference)		
Living	Living alone	88	0.68	0.25–1.88		
Conditions	Cohabiting	27	1	(Reference)		
Care	Family participates	41	3.11	1.15–8.44	3.17	1.12–9.00
	Family does not participate	69	1	(Reference)	1	(Reference)
	Home Help^1^	68	0.31	0.11–0.89		
	No Home Help	35	1	(Reference)		
Mobility	Reduced ^2^	58	0.51	0.22–1.17		
	Not reduced	57	1	(Reference)		
Cognitive function	Reduced ^3^	38	0.52	0.23–1.22		
	Not reduced	77	1	(Reference)		
ADL dependence	Yes, severe (group 3)^4^	23	0.20	0.08–0.53	0.21	0.08–0.59
	None, or not severe (Groups 1–2)^5^	92	1	(Reference)	1	(Reference)
Goodness of fit, Pearsson				0.58		

^1 ^Not included, see explanation in the text, ^2 ^Is unable to move about in immediate surroundings, ^3 ^Difficulty in knowing day of week, finding the way home and/or recognising relatives/caregivers, ^4 ^Needs help to eat, ^5 ^Independent or needs help with cleaning, shopping, transport, bathing, cooking and/or dressing, but not eating.
